# Reviewer acknowledgement 2012

**DOI:** 10.1186/1748-7161-8-5

**Published:** 2013-03-22

**Authors:** Theodoros B Grivas

**Affiliations:** 1 "Tzanio" General Hospital of Piraeus, Piraeus, Greece

## Abstract

**Contributing reviewers:**

The Editor-in-Chief of *Scoliosis* would like to thank all our reviewers who have contributed to the journal in Volume 7 (2012).

## 

Emre Acaroglu

Turkey

Clayton Adam

Australia

Behrooz Akbarnia

United States of America

Carl-Eric Aubin

Canada

Lorenzo Aulisa

Italy

Keith Bagnall

Canada

Saumyajit Basu

India

Marie Beausejour

Canada

Rowan Berkowitz

South Africa

Josette Bettany-Saltikov

United Kingdom

Jens Ivar Brox

Norway

Richard Burwell

United Kingdom

Rene Marten Castelein

Netherlands

Jack Cheng

Hong Kong

Nachiappan Chockalingam

United Kingdom

Winnie Chu

China

Peter Dangerfield

United Kingdom

Jean Claude De Mauroy

France

Sabrina Donzelli

Italy

Jean Dubousset

France

Ferran Escalada

Spain

Jeremy Fairbank

United Kingdom

Charla Fischer

United States of America

Azmi Hamzaoglu

Turkey

Martha Hawes

United States of America

Manabu Ito

Japan

Donald Katz

United States of America

Eustathios Kenanidis

Greece

Patrick Knott

United States of America

Tomasz Kotwicki

Poland

Virginie Lafage

United States of America

Lawrence Lenke

United States of America

Keith Luk

China

Jean-Marc Mac-Thiong

Canada

Toru Maruyama

Japan

Jwalant Mehta

United Kingdom

Stefano Negrini

Italy

Joseph O'Brien

United States of America

James Ogilvie

United States of America

Acke Ohlin

Sweden

Harry Opsimos

United States of America

Peter John Papantoniou

Australia

Vikas Patel

United States of America

Petros Patias

Greece

Nigel Price

United States of America

Gerald Quan

Australia

Manuel Rigo

Spain

Michele Romano

Italy

Jose Miguel Sanchez Marquez

Spain

Alpaslan Senkoylu

Turkey

Konstantinos Soultanis

Greece

David Speers

United States of America

Luke Stikeleather

United States of America

Piet van Loon

Netherlands

Marian H Wade

United States of America

Yan Wang

China

Hans-Rudolf Weiss

Germany

Adam Wollowick

United States of America

Man-Sang Wong

Hong Kong

James Wynne

United States of America

Fabio Zaina

Italy

Daniel Zarzycki

Poland

